# Supplementary material for: Chromothripsis during telomere crisis is independent of NHEJ, and consistent with a replicative origin
Source: Genome Res. 2019 May;29(5):737–49. doi: 10.1101/gr.240705.118 (PMC6499312; doi:10.1101/gr.240705.118)
Supplement: Supplemental Material [file supp_gr.240705.118_Supplemental_file_1.zip › contigs/annotated_contigs/DB75/contig.2.DB75_length_575_mean_cov_4.92869565217.docx]

**DB75_length_575_mean_cov_4.92869565217**

```
ATGTAAGCCAAAAAAATCTAAATACAATTTTATATTTGACAGTGCTTCTTGTATAATTTTAACATGCCAAATAAGCCAAGTATGTCTCT
 >chrX:113235201-113235520 - E=3e-177
CTTGGCCTTCTGAAAAGTTAGTTTTAGGCCAAGAGACTGAATTTACAATTTGATTTTTAGAAAGTTTGGCAAATATCAAAGGTTCAAAA

CACTCGATACCACAAAATGGAATTACAGGTCACTCACTGTGATGGTTAATACTGAGCATCGACTTGATTGGATTGAAGGATACAAAGTA

ATGATCCTGGGTGTGTCTGTGAGGGTGTGGCCAAAAGAGATTAACATTTGAG|GTAACAAAAAT|TGTGTGTGTGTGTGTATATATATA
                                                                >chrX:89601031-89601276
TATATATATATATACTGTTTAGCCATATACAGCCATGTGCCATATGACAACATTTTCATCAAGGTCAGACTGATGTGGTAGCTTTCATA
 + E=1e-126
ACATTGTAGCCCAACACAATGGGCTTATTCTGTTTAGATTTATTCTGTTTAGATATCTTGCATACACAAGTACCATTACATTACAATTG

CCTACAGCATTCAATGCAGTAACCTGCTTACCAAGTTTGTAGA
```
